# Management, Levels of Support, Quality of Life, and Social Inclusion in Parkinson's Disease: Interventions, Innovation, and Practice Development

**DOI:** 10.1155/2021/4681251

**Published:** 2021-01-29

**Authors:** Mari Carmen Portillo, Anita Haahr, M. Victoria Navarta-Sánchez

**Affiliations:** ^1^University of Southampton, School of Health Sciences, NIHR ARC Wessex, Southampton, UK; ^2^Department of Public Health, Aarhus University, Aarhus, Denmark; ^3^Autonomous University of Madrid, Faculty of Medicine, Nursing Department, Madrid, Spain

Parkinson's disease (PD) is the second most common neurodegenerative disorder worldwide with a prevalence of 1% in populations over 60 years of age in developed countries and affecting 10 million people worldwide [1, 2]. Compared to other neurological conditions, PD has had the highest growth in terms of disability, death, and prevalence, and it is estimated to increase to 13 million by 2040 [3].

The experience of living with PD varies from person to person and captures complex issues that go beyond biomedical management and require a comprehensive approach involving the family, overcoming health inequalities, and ensuring sustainability in policy and health/social care. In line with this, in this special issue, we aimed to explore aspects related to PD management and innovation from a health and social care perspective. A total of 16 manuscripts were submitted, from which 9 were accepted for publication (1 review and 8 original studies). Accepted manuscripts covered a wide variety of topics such as the assessment and management of motor/nonmotor and cognitive symptoms and their impact on patients' and carers' QoL, proposals for integrated care models, and the psychosocial impact of PD and related interventions.

The diversity of PD symptoms and impairment, progressive nature, and psychosocial impact lead to a burden and a major decrease in the QoL not only for patients but equally for carers [4–6]. Therefore, targeted assessments to register the complexity of PD become an important asset for PD management as highlighted in four papers in this special issue. In a cross-sectional study, Sun and colleagues focused on the clinical assessment of the development of motor symptoms and the use of levodopa for their management. Based on their results, they suggested—in the case of levodopa-induced motor complications—to consider delaying or reducing the dose of levodopa. Pereiro et al. explored the connections between the stages of PD with the presence of cognitive and motor skills, being more important maintaining both skills in comparison with fluency and single motor skills. This revealed relevant implications for clinical practice to determine PD progression and the need to introduce more adequate assessments in research with expected benefits in the evaluation of some interventions. However, we must remain cautious interpreting these findings considering the small scale of the research reported. Jenny et al. also undertook a quantitative study determining the impact of cognitive complaints on QoL from the patients' perspective. Based on a survey of 46 patients, they compared subjective memory complaints with QoL, using the PDQ 39 instrument. Their main hypothesis that subjective memory complaints could be associated with QoL was dismissed. Notwithstanding, they show an association between subjective memory complaints and cognitive QoL, and likewise, they found an association with anxiety and depression. Jenny et al. also addressed the need for subjective memory complaint validated tools to enhance accuracy in referrals to specialized PD care. Finally, Klietz et al. looked at the associations/correlations between motor and nonmotor and cognitive symptoms and health-related QoL and carer burden in advanced PD. Main findings indicated that motor symptoms are clearly associated with the carer's burden more than other symptoms in PD, and all motor and nonmotor have a similar impact on health-related QoL of patients. Impaired attentional functioning in patients with PD also affected carers in equal proportion. This clearly highlights a clinical priority, which is to understand PD symptomatology and draw management plans including the carer's needs. Integrating instruments that contemplate the needs of both the person with PD and the carer in routine care could result in more optimal healthcare utilization without sacrificing QoL and economic costs. This could also ensure more effective risk stratification and early identification of people with higher needs for more complex care coordination and with high or low risk for poor self-management, facilitating the referral process and points of support for the family unit.

Therefore, the impact of PD on patients and carers as a unit and how they access healthcare services and resources in the community should not be overseen [4]. Being a caregiver to a person with PD often causes a significant strain on the family member, and studies show that the everyday lives of caregivers are often negatively affected, including a decrease in QoL and psychiatric symptoms [7, 8]. In this special issue, two papers explored carers' psychosocial adjustment to PD and QoL and their participation in psychosocial interventions. The paper by Lopes Dos Santos and colleagues in this special issue reports on an observational comparative study of the psychosocial adjustment of caregivers of persons with either dementia or PD. Overall, they reported the perceived psychosocial adjustment to the condition to be good, which is somewhat different from the reports in other studies. Lopes Dos Santos et al. did find caregivers to people with dementia to be more vulnerable than the other group, also reporting lower satisfaction with life than caregivers to people with PD. In their study, the participants with PD did not have dementia, which, as the authors reflected, calls for special attention because dementia is common in PD. Thus, as the people with PD included in this study had no cognitive decline, this could mean that caregivers of people with PD with cognitive decline could be more vulnerable.

Additionally, Prado et al. presented an innovative point of view about the level of participation of carers in research interventions or programs that are normally designed for people with PD through a mixed-methods research design. The authors concretely looked at psychosocial interventions like dancing, which have become quite popular nowadays to improve gait, coordination, and socialization [9–11]. Among the reasons to attend the interventions, participants mentioned being together with the patient, socializing, increasing physical activity, and using it as respite care. However, a negative aspect of participating in these activities for the person with PD was the decrease in free time for the carers themselves. Prado et al. suggested that carers could be encouraged to attend 2/3 of the activities of the program and leave 1/3 of free time for themselves as an ideal balance between social participation and relief from their caring role.

Person-centred and integrated approaches are gaining importance to manage complex needs in LTCs. Recent policy initiatives [12, 13] promoting integrated care have achieved positive outcomes for conditions such as cancer, cardiovascular diseases, and diabetes. However, only in regard to PD, integrated care is at an early promising start [14–16] with lack of answers about how this would work for the progressive nature of PD [17]. Although PD requires less fragmented management across levels of health care, between health and social care systems, and among other complementary systems of support in the community [3, 13], the uncertainty, diversity, and instability of the symptomatology of PD could jeopardize the sustainability of coordinated attempts to manage the condition and its holistic consequences [14]. The European Parkinson's Disease Association (EPDA) has reported large gaps in care delivery for PD [2], especially in rural areas. Integrated care initiatives for PD should put the emphasis not only on symptom management but also on coping skills and psychosocial adjustment to illness. Nevertheless, as stated above, this cannot be achieved without appropriate instruments to help professionals assess and understand individual circumstances, available levels of social support, and indirect costs of living with PD. In this special issue, three papers provided further understanding about integrated care models for people with PD and carers. Muñoz and colleagues undertook a quantitative study using surveys to explore the value of education and the interdisciplinary approach for improving well-being and QoL in people with PD and their informal caregivers in the community context. These authors presented the first multidisciplinary initiative for people with PD and their informal caregivers in a low-middle-income country (Colombia). Their approaches consisted of three types of sessions: educational sessions, cognitive or physical rehabilitation, and leisure activities working with people with PD, informal caregivers, and professionals, who reported improvements in QoL and sense of burden especially in carers. In line with these works with less advantaged populations, Iansek and Danoudis carried out a quantitative descriptive study with a survey to explore patients' healthcare experiences of inequalities in access to health care comparing two different cohorts of people with PD living in rural Victoria. One cohort received comprehensive care and the other received standard rural care, which is of special relevance considering the difficulties in access to specialized services that can be encountered in rural areas [18]. Findings from the patients' surveys showed that patients who received comprehensive care had better key healthcare experiences and QoL and less impairment and disability. The provision of individualized information was considered an essential component of the programme. Finally, Tenison et al. underlined the need for an integrated care model for PD, especially when there is evidence of integrated care models for other LTCs [12, 13]. Based on a review and analysis of the literature and current practice/policy, they proposed a logic integrated model that will be tested in the UK and the Netherlands and has 5 components: personalized care management, education and empowerment of patients and carers, empowerment of health professionals, population health approach, and use of technology to support this. The next phase of the study will consist of testing the new model impact on QoL and measuring its cost-effectiveness through an RCT looking at levels of acceptance and psychosocial adjustment to the condition [19]. Additionally, the model also includes individually tailored information, which may be difficult to achieve without an adequate mobilization of resources and levels of care.

This special issue has shed some light on recent findings and/or programmes that could result in benefits for people with PD and family carers in relation to psychosocial adjustment, integrated care, QoL, and targeted assessments. Sustainable and integrated health-supporting environments are essential for people living with PD to ensure patients' and families' social inclusion and access to care. Implementation plans for innovation, technology, and care pathways for PD require a complex system of support, which starts from involving the patient and their family by establishing dialogue between agents, disciplines, and levels of care.

Further research is still needed in relation to integrated care for PD looking at multidisciplinary and multilevel interventions to equally empower people with PD and their carers in the community context. Evidence from randomized controlled trials and similar studies is necessary to evaluate this type of intervention, especially in rural areas where people with PD may encounter poorer access to specialized care. Moreover, the availability of more targeted and valid assessment tools in clinical practice could result in better identification of physical and nonphysical needs, adjustment to the condition, and management of daily life for people with PD and carers. Finally, further evaluation of new integrated care models is essential to ensure their implementation and transferability in health systems and clinical practice [20], optimising the resource availability for people with PD and carers and with a special emphasis on the community and disadvantaged populations.

Some existing research across Europe [21] leaves a door open for research collaborations that could develop further understanding of what is meant by integrated and multisectoral care models, how this could be evaluated and adapted to different care settings and health systems, and what role patient associations, people with PD, and carers could play in the clinical implementation of such models [21].

*Mari Carmen Portillo* *Anita Haahr* *Victoria Navarta*
